# A multi-threaded approach to genotype pattern mining for detecting digenic disease genes

**DOI:** 10.3389/fgene.2023.1222517

**Published:** 2023-08-24

**Authors:** Qingrun Zhang, Muskan Bhatia, Taesung Park, Jurg Ott

**Affiliations:** ^1^ Department of Mathematics and Statistics, University of Calgary, Calgary, AB, Canada; ^2^ Department of Biochemistry and Molecular Biology, University of Calgary, Calgary, AB, Canada; ^3^ Amity Institute of Biotechnology, Amity University Madhya Pradesh, Gwalior, India; ^4^ Department of Statistics, Seoul National University, Seoul, Republic of Korea; ^5^ Laboratory of Statistical Genetics, Rockefeller University, New York, NY, United States

**Keywords:** digenic trait, genetic association, genotype pair, genetic variant, single-nucleotide polymorphism

## Abstract

To locate disease-causing DNA variants on the human gene map, the customary approach has been to carry out a genome-wide association study for one variant after another by testing for genotype frequency differences between individuals affected and unaffected with disease. So-called digenic traits are due to the combined effects of two variants, often on different chromosomes, while individual variants may have little or no effect on disease. Machine learning approaches have been developed to find variant pairs underlying digenic traits. However, many of these methods have large memory requirements so that only small datasets can be analyzed. The increasing availability of desktop computers with large numbers of processors and suitable programming to distribute the workload evenly over all processors in a machine make a new and relatively straightforward approach possible, that is, to evaluate all existing variant and genotype pairs for disease association. We present a prototype of such a method with two components, *Vpairs* and *Gpairs*, and demonstrate its advantages over existing implementations of such well-known algorithms as *Apriori* and *FP-growth*. We apply these methods to published case-control datasets on age-related macular degeneration and Parkinson disease and construct an ROC curve for a large set of genotype patterns.

## 1 Introduction

Many heritable traits are governed by a single DNA variant (single-nucleotide polymorphism, SNP) or a single gene, for example, cystic fibrosis (Tsui et al., 1985). Such disease variants can generally be detected in the course of a genome-wide association study (GWAS), where for one variant after another, genotype frequencies are compared between cases (affected by disease) and control individuals. Various statistical significance tests are in common use and have been implemented, for example, in *plink* (Chang et al., 2015), which also serves as a standard for representing and storing genetic data. While genetic origins of many single-gene (mendelian) disorders have been elucidated, there are genetic traits that are under the control of the action and interaction of large numbers of genes, which evidently are difficult to pinpoint individually. A prime example of such polygenic traits is schizophrenia (Purcell et al., 2009; Trifu et al., 2020). In between these extremes are so-called digenic traits, which are influenced by two mutant variants, often on different chromosomes, while one such variant alone does not cause disease (Schaffer, 2013).

Various statistical and machine learning approaches have been applied to find pairs of genes or of DNA variants underlying digenic traits (Papadimitriou et al., 2019; Okazaki and Ott, 2022; Ott and Park, 2022). Methods based on market-basket analysis (frequent pattern mining, FPM) apply some form of the *Apriori* principle (Agrawal and Srikant, 1994), which focuses on common patterns and allows skipping large numbers of infrequent patterns.

As demonstrated in the *Results* section, many FPM approaches require large amounts of computer memory (RAM). An exception is our own method, *AprioriGWAS* (Zhang et al., 2014), which applies the HDF5 file format to avoid excessive RAM consumption, but this program is difficult to use generally although its executable code is available online, https://github.com/Qingrun/AprioriGWAS. The restriction to frequent patterns in cases has clear practical and computational advantages, but patterns that are rare in cases yet frequent in controls may be equally valuable. Most of the methods for genotype pattern mining mentioned in our previous overview (Okazaki et al., 2021) can only be applied to small datasets as they require too many computer resources for larger datasets. Other methods (Lee et al., 2014; Shang et al., 2016) have been described as being powerful and capable of handling large numbers of variants, but no software is available for them.

Here, we are proposing to evaluate all possible pairs (patterns) of variants and genotypes and test each pattern for frequency differences in cases and controls. Such an approach has until recently been considered impractical and searches have been restricted, for example, to biologically plausible patterns (Lee et al., 2018). However, current workstations contain relatively large numbers of CPUs (threads), and suitable programming allows for the processing of all variant and genotype patterns in medium-sized datasets by distributing the workload over CPUs. We present a prototype of such an approach and make software freely available that can run on general-purpose workstations. It is written in Pascal (https://www.freepascal.org/) and the executable code runs in Windows or Linux.

## 2 Methods

In our new approach to mining variant and genotype pairs (patterns), rather than relying on the *Apriori* property to eliminate infrequent patterns, we aim to evaluate all suitable patterns and do this by harnessing the availability of multiple CPUs in a workstation. The principle adopted is quite simple. For *N* variants, we assign a sequential number to each variant pair: 1, 2, … , *M*, with *M* = *N*(*N*–1)/2. Numbering variant pairs is conveniently carried out numerically in two nested loops, with one loop ranging from *i* = 1 through (*N*–1) and the other ranging from *i* + 1 through *N*. With *t* available CPUs, each CPU is assigned to work on *M*/*t* variant patterns. Thus, all but possibly the last CPU will work on the same number of variant patterns. This numbering scheme can also allow to exclude variant pairs when both participating variants reside on the same chromosome if so desired. Some approaches working with pairs of variants (Chang et al., 2015) restrict the two variants in a pair from being too close to each other in order to avoid any disturbing effects of linkage disequilibrium. Here we address this potential concern by letting users decide whether to exclude variants in a pair when they reside on the same chromosome.

Now we embark on two different avenues, one to work on variant pairs and the other to consider all possible genotype pairs.

### 2.1 Variant pairs

As shown in Table 1, each variant considered has two alleles, A and B, and corresponding three genotypes numbered *1* = A/A, *2* = A/B, and *3* = B/B, so a given pair of variants comprises nine genotype pairs, corresponding to eight degrees of freedom (df), of which two represent main effects for each of the two variants, with four df representing interaction effects between the two variants. Single-variant analysis may reveal whether a given variant by itself (its main effect) leads to a difference in genotype frequencies between cases and controls. Thus, for a pair of variants, what remains to be tested is whether the interaction between the two variants in a pair is different between cases and controls. Various approaches have been proposed to address this question, two of which have been implemented in *plink* (Wan et al., 2010; Ueki and Cordell, 2012). Here, we propose a likelihood ratio test of the null hypothesis, *H*_0_, that there is no difference in interaction between cases and controls. Considering the alternative hypothesis, we compute a standard interaction chi-square for cases and controls each [note that these statistics need to be computed as likelihood ratio chi-squares, G^2^ (Agresti, 2013)], that is, G^2^_case_ and G^2^_ctrl_ with 4 df each, where G^2^ = 2 Σ *n*_j_ log(*n*_j_/*e*_j_), and *n*_j_ and *e*_j_ are respective observed and expected numbers of observations in the *j*-th cell of the table Given *H*_0_, we combine cases and controls and compute a chi-square for the combined data as G^2^_case+ctrl_. Then, the appropriate test statistic is given by *C* = G^2^_case_ + G^2^_ctrl_–G^2^_case+ctrl_, which under *H*_0_ follows a chi-square distribution with 4 df. This interaction test has been implemented in a Pascal program, *Vpairs*, with executable code available online (https://www.jurgott.org/linkage/GPM.html) for Windows and Linux.

**TABLE 1:** Layout of case-control genotypes for a given pair of variants. Labels *1*, *2*, and *3* refer to genotypes with *1* = AA, *2* = AB, and *3* = BB. The numbers in the body of the table represent individuals with given genotype pairs at the most significant variant pair for the AMD dataset (Section 3.1.1).

Cases	rs9298846			Controls	rs9298846		
rs994542	*1*	*2*	*3*	rs994542	*1*	*2*	*3*
*1*	1	0	10	*1*	2	11	0
*2*	5	26	28	*2*	4	10	4
*3*	5	16	5	*3*	0	4	15

Large values of *C* represent evidence for an interaction difference between cases and controls, and we carry out this test for all *M* variant pairs. Because of the computational burden, we do not consider permutation testing at this point, but instead rely on Bonferroni-corrected *p*-values, *p*_B_ = min(1, *p* × *M*_t_), where *p* is the empirical significance level associated with *C*, and the number of tests, *M*_t_, is given by *M* minus the number of variant pairs in which any expected number in the chi-square calculations falls below a critical limit such as 1 (user-defined); if desired, a variant pair may also be disregarded when its two variants are on the same chromosome. For the best results, a researcher may then want to compute odds ratios for each of the nine genotype pairs to see which one(s) drive the large *C* value. For a given genotype pair, *X*, as shown in Table 2, we compute an odds ratio as OR = (*a* + ½)(*d* + ½)/[(*b* + ½)(*c* + ½)] (Agresti, 2007). As we are interested in genotype pairs that are more common or less common in cases than controls, we will work with OR’ = max(OR, 1/OR).

**TABLE 2:** Numbers *a*, *b*, *c*, and *d* of individuals by phenotype and presence/absence of a genotype pattern, *X*. The actual numbers refer to pattern, *X* = (*1*, *2*) for variant pair (rs994542, rs9298846) in the AMD dataset. There is a complete lack of *X* in cases while eleven control individuals carry this genotype pattern.

*Phenotype*	*X* present	*X* absent	*Total*
Cases	*a* = 0	*b* = 96	96
Controls	*c* = 11	*d* = 39	50
sum	11	135	146

### 2.2 Genotype pairs

In analogy to many FPM approaches, we also implemented a search of all suitable genotype pairs. This procedure starts at a given pair of variants. For each of its nine genotype pairs, we only consider pairs with known genotypes and compute a 2 × 2 table of known genotypes as shown in Table 2. Values of *a*, *b*, *c*, and *d* represent numbers of individuals (cases or controls, carrying or not carrying a given genotype pattern, *X*). As is customary in FPM methods, we call (*a* + *c*) the *support* for pattern *X*, while *a*/(*a* + *c*) is referred to as *confidence* (Agrawal and Srikant, 1994), usually expressed as a percentage, which in statistics is known as the predictive value. A given variant pair furnishes up to nine genotype pairs, and all suitable genotype pairs are found by evaluating all variant pairs and the genotype pairs contained in each.

Users generally are only interested in patterns (genotype pairs) with some minimum support, that is, patterns occurring with frequencies smaller than a minimum specified by the user will not be tested (Borgelt, 2012). The number of tested patterns will form the basis for Bonferroni-corrected *p*-values. A minimum confidence may also be specified to restrict patterns to a set with high predictive values.

Each genotype pair furnishes a 2 × 2 table like the one shown in Table 2, and the number of tests is determined in analogy to *M*_t_ in section 2.1. Thus, the number of genotype pairs is roughly nine times larger than the number of variant pairs. For each of the 2 × 2 tables of phenotype versus presence of a given genotype pattern, we consider two test statistics, Pearson chi-square and Fisher’s exact test.

There are two main advantages to our complete enumeration of all suitable genotype patterns. First, in the course of finding genotype patterns, we don’t need to keep track of previously found patterns as is the case with *Apriori*-type approaches. Consequently, memory requirements for our methods are rather small and, as demonstrated below, we can easily handle datasets for which *Apriori*-type approaches run out of memory. Secondly, our technique allows for the proper handling of missing data. To appreciate this, assume that a machine learning approach has identified a given pattern, *X*, in a number of cases and controls, so the “*X present*” column in Table 2 has been filled. To also populate the “*X absent*” column, the approach generally taken must be subtraction from the totals. For example, to find the number of cases without *X*, *b* is obtained as the total number of cases minus *a*. However, this value *b* also contains unknown genotypes unless a dataset is completely free of missing genotypes, which is rare. In our approach, for a given pairs of variants, we initially work with a 4 × 4 table of genotypes, where “missing” is treated as a fourth “genotype.” Then we focus on the 3 × 3 subtable of known genotypes and, in Table 2, find the *Totals* as the total number of cases or controls with known genotypes. Our complete enumeration of genotype patterns furnishes an exact count of all patterns with non-missing genotypes, which will serve as the basis for Bonferroni-corrected *p*-values.

## 3 Results

We applied our implementations of *Vpairs* and *Gpairs* to several published datasets. Generally, it is advisable to run *Vpairs* first and then follow up with *Gpairs* or a utility program, *pairSNPs*, to investigate whether any of the nine genotype pairs in a given variant pair show an unusually large odds ratio (OR’), which is a common measure of effect size. As the number of variant pairs is much smaller than the number of genotype pairs, Bonferroni correction may be more efficient for variant pairs than genotype pairs.

All analyses below were run on Linux (Kubuntu) machines. In our experience, some jobs were able to run in Linux with 32 GB yet ran out of memory in Windows with 64 GB, which may be due to differences in efficiencies of memory managers in Windows and Linux.

### 3.1 AMD dataset

Our previously published dataset on age-related macular degeneration is available in *plink* format in the program package, https://www.jurgott.org/linkage/GPM.html. The dataset comprises 96 cases and 50 controls, each genotyped for 103,611 autosomal variants. Single-variant analysis in *plink* by the trend test furnished two significant variants, rs380390 and rs1329428 (Klein et al., 2005), both on chromosome 1, with respective significance levels of *p*_B_ = 0.0322 and 0.0900 (100,000 permutation replicates furnished respective significance levels of 0.0117 and 0.0361). To avoid finding many variant and genotype pairs involving the two significant variants, they were omitted from subsequent pattern analysis. This slightly reduced dataset was run on a Linux machine with 512 GB of RAM and 8 CPUs, of which 5 were dedicated to the pattern analyses.

Genotype patterns involve main and interaction effects (Okazaki et al., 2021) so that variants with strong main effects tend to show up in large numbers of significant genotype patterns (*Gpairs* algorithm), but this phenomenon is not expected when we apply *Vpairs* as this algorithm is designed to specifically test for interaction differences between cases and controls, irrespective of main effects of variants.

#### 3.1.1 AMD variant pairs

We ran the AMD dataset with our *Vpairs* program, requiring that the two variants in a pair reside on different chromosomes. This resulted in a total of 5,050,626,692 variant pairs. Due to the requirement of expected numbers of observations of at least 1 in each cell of the 3 × 3 table of genotypes of a given variant pair, only 294,643,816 variant pairs were analyzed, which is also the number *M*_t_ of tests used for calculating Bonferroni-corrected *p*-values. As shown below, we compared our results with the *joint-effects* method (Ueki and Cordell, 2012) implemented in *plink*.

*Vpairs* ran in 25 min and furnished a significant variant pair, rs994542 on chromosome 6 and rs9298846 on chromosome 9, *p*_B_ = 0.0380. This variant pair was also reported by previous authors (Shang et al., 2016) as the most prominent result in their application of two search strategies for interacting variants in the AMD data.

For the most significant variant pair, Table 1 shows numbers of individuals by phenotype and genotype at each of the two implicated variants. The most prominent result in terms of odds ratios is the complete lack of (*1*, *2*) genotypes in cases while eleven control individuals carry this genotype pattern (OR for cases = 0.0178, 1/OR = 56.19). For this genotype pattern, *X* = (*1*, *2*), Table 2 shows numbers of individuals by phenotype and presence/absence of *X*. Further discussion of such genotype pattern tables will be provided in the next section.

The *plink* program ran the *joint-effects* method in 9 min but did not result in a variant pair with *p*_B_ < 1, perhaps partially because the number of tests, *M*_t_ = 5,367,360,636, greatly exceeded that in the analysis by *Vpairs* (the *joint-effects* analysis considers all variant pairs, also pairs consisting of two variants on the same chromosome). The best variant pair reported by the *joint-effects* analysis was rs4128236 on chromosome 3 and rs10508482 on chromosome 10. The latter variant is a genic upstream transcript variant in the FAM107B gene and has been reported to be involved in gastric cancer (Guo et al., 2017). This variant pair was not found among the best 35,000 variant pairs reported by *Vpairs*. Conversely, the best variant pair in *Vpairs* was not found among the best 100,000 variant pairs identified by the *joint-effects* method even though that variant pair had been confirmed by several analysis methods (Shang et al., 2016). Thus, applying multiple analysis methods and interpreting different results seems to be a good strategy.

In the trend test of the AMD dataset missing the two variants with strongest single-variant main effects, the best single variant, rs10272438 on chromosome 7, showed *p*_B_ = 1 (100,000 permutation datasets furnished an empirical significance level of 0.6034), yet, as shown above, the same dataset provided very significant results for *pairs* of variants. This phenomenon may well be a rather common occurrence once we analyze many datasets for pairs of variants and genotypes.

#### 3.1.2 AMD genotype patterns

To test whether genotype pattern frequencies are different in cases than controls, we consider an exhaustive list of variant pairs as described in the previous section and, for a given variant pair, test each of the 3 × 3 = 9 genotype pairs. Each genotype pair will furnish a 2 × 2 table (Table 2), for which we carry out one of two tests, either 1) a chi-square test or 2) Fisher’s exact test, where the latter may be applied in one of three ways, as a one-sided test for patterns being more frequent in cases than controls (F2 test), a one-sided test for patterns in controls being more frequent than in cases (F1 test), and as a two-sided (F3) test (Irwin, 1935; Agresti, 2013). Ranking genotype pairs resulting from these tests will somewhat depend on the test statistic applied. We may also rank genotype pairs by the OR’ metric, which is independent of test statistics as it only depends on the four numbers of observations shown in Table 2. In contrast to the chi-square test, there is no need for the Fisher test to impose a minimum number of expected observations in 2 × 2 tables as the resulting *p*-values are always accurate.

As mentioned above, the *Vpairs* analysis for the AMD data has identified a significant variant pair (*p*_B_ = 0.0380). For this variant pair, the genotype pair with largest OR’ value of 56.2 was (*1*, *2*), with genotype *1* being at variant rs994542 and genotype *2* at variant rs9298846. On the other hand, analyzing all pairs of genotypes by the *Gpairs* program (with minimum support of 10 and no restriction on confidence) took 88.3 min for the chi-square test and 125.1 min for the two-sided Fisher test (F3). None of the two tests furnished *p*_B_ values less than 1, possibly because of the large increase in numbers of tests done as compared with the variant pair analysis. Somewhat surprisingly, the genotype pair (*1*, *2*) identified above was not among the top few genotype pairs furnished by the *Gpairs* algorithm. In fact, that “favorite” genotype pair had rank 19,148 in the chi-square test, 23,820 in the Fisher test, and 2,004 on the OR’ scale. That is, the *Gpairs* analysis identified at least 2,004 stronger genotype pairs than those extracted from the variant pair analysis, and each of these 2,004 genotype pairs refers to variants on different chromosomes. Thus, while the *Vpairs* algorithm furnishes one test statistic reflecting the average effect over nine genotype pairs, which may mask strong effects of single genotype pairs, the *Gpairs* algorithm is capable of much finer detection of genotype pairs underlying digenic traits. Whereas various genes underlying AMD have been reported in the literature, this trait has not previously been considered to be digenic, but evidently there exist many correlations among genotypes, even in variants on different chromosomes, that are related to disease.

To compare our exhaustive search of genotype pairs with conventional FPM algorithms, we ran the AMD dataset also with an implementation of an algorithm, *Mining the Top-K Class Association Rules* (Top-K), which is more efficient than *Apriori* but still uses much memory (RAM) (https://www.philippe-fournier-viger.com/spmf/). *Gpairs* was run in 5 CPUs while *Top-K* as a java program used one CPU but occasionally up to 8 CPUs. *Gpairs* and the *Top-K* algorithms furnished the following respective runtime characteristics: 2.2 and 403.8 GB of RAM used, and execution times of 32 and 197 min. We also tested an implementation of the *FP-growth* pattern mining algorithm (Borgelt, 2005) but it ran out of memory before completion (details not shown). Clearly, *Gpairs* is capable of analyzing small datasets efficiently and quickly and is suitable for larger datasets although at the expense of longer runtimes. An example is provided in Section 3.2 on Parkinson Disease.

**FIGURE 1:**
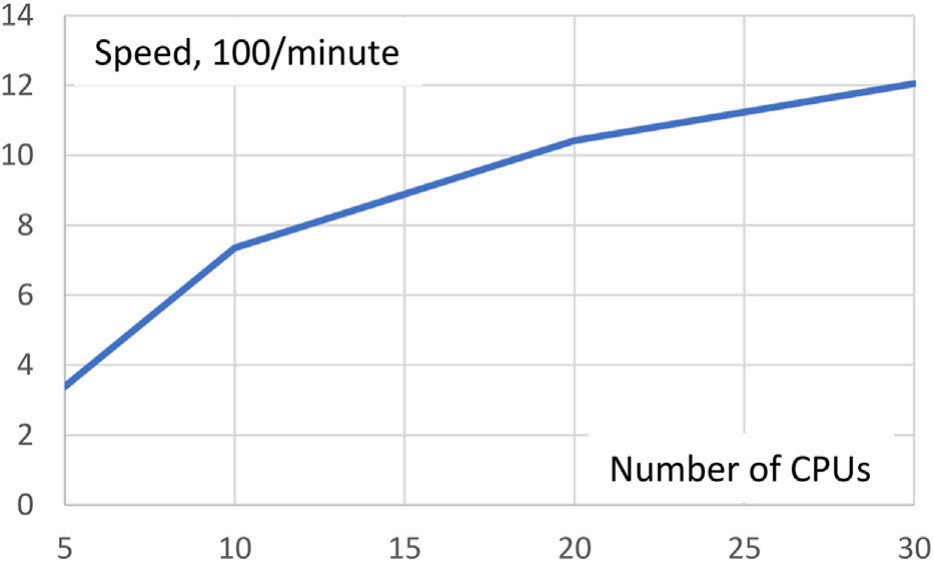
Execution speed analysis of the AMD dataset depending on the number of CPUs invoked. Doubling the number of CPUs from 5 to 10 more than doubles the speed, but a further doubling from 10 to 20 CPUs only increases speed by 42%.

As mentioned above, *Gpairs* equally distributes the number of genotype pairs to be processed over all CPUs invoked. The more CPUs are used the fewer genotype pairs each CPU has to process, but management overhead of the CPUs will increase somewhat. To see the net effect of these two opposing forces, we ran the AMD data with *Gpairs* multiple times for different numbers of CPUs in a Linux machine with 36 CPUs. Execution times for 5, 10, 20, and 30 CPUs were 29.5, 13.6, 9.6, and 8.3 min, respectively. Expressed in terms of speed, 100/minutes, Figure 1 shows resulting increases in speed. Clearly, the increase in efficiency is greatest at small numbers of CPUs. Doubling the number of CPUs from 5 to 10 more than doubles the speed, but a further doubling from 10 to 20 CPUs only increases speed by 42%.

### 3.2 PD dataset

A case-control dataset on Parkinson Disease (PD) (Fung et al., 2006) was used to demonstrate application of our methods to a mid-sized dataset. After standard data cleaning, it contained 270 cases and 271 controls, each genotyped for 379,502 variants. Using 30 CPUs in a desktop Linux machine, the *Vpairs* program evaluated 68 × 10^9^ pairs of variants in 16.1 h (each of the two variants in a pair was on different chromosomes). The best pair, rs1323114 on chromosome 13 and rs7188399 on chromosome 16, showed *p*_B_ = 0.2701, but none of these two variants are in a gene region or showed up in literature searches. One genotype pair, (*1*, *1*), stood out with an odds ratio of OR’ = 51.4: That genotype pair occurred in 23 controls and was completely absent in cases.

The *Gpairs* program ran for 14.5 h and applied Fisher’s two-sided test to each of 583 × 10^9^ genotype pairs, selecting pairs with minimum support of 20 and no restriction on confidence. None of the resulting genotype pairs showed *p*_B_ < 1. On the Fisher test *p*-value scale, the genotype pair (*1*, *1*) extracted from the *Vpairs* analysis had rank 960, that is, 960 genotype pairs had a Fisher test *p*-value equal to or smaller than the genotype pair (*1*, *1*) in the best variant pair obtained by *Vpairs*. The genotype pair with overall smallest Fisher test significance level occurred in variant pair rs13179395 on chromosome 5 (genic downstream transcript variant in the LINC00461 gene, which produces non-coding RNAs that may predominantly be expressed in the brain) and rs4356177 on chromosome 10 (not in a gene). The relevant genotype pair, a double heterozygote (*2*, *2*), occurred in 33 controls but not in any of the cases.

## 4 Sensitivity and specificity

Our approach implements a pattern search algorithm to find genotype pairs (patterns) capable of discriminating between cases and controls. For each of a potentially very large number of patterns, we generate a 2 × 2 table as shown in Table 2 and apply Fisher’s exact test. A single pattern may occur only in a few cases or controls so that only a few cells of Table 2 will be sparsely populated, and no meaningful result is obtained. To evaluate large numbers of patterns and their associated 2 × 2 tables, we view occurrence of a pattern in an individual as an indicator of that individual’s phenotype (case or control for test type F2 or F1, respectively) so that the two columns in Table 2 may be seen as “predicting presence of phenotype” and “predicting absence of phenotype.” In other words, we turn Table 2 into a decision matrix (Nahm, 2022), in which the number *a* of individuals represent true positives (TP) as the predicted phenotype agrees with the known phenotype. On the other hand, *d* individuals are true negatives (TN), *c* individuals are false positives (FP; they are predicted to be cases while they are known to be controls), and *b* individuals are false negatives (FN). The rate of true positives, TPR = *a*/(*a* + *b*), is known as sensitivity or power, and the true negative rate, TNR = *d*/(*c* + *d*), is called specificity. The relationship between these quantities is generally expressed as a graph called an ROC curve, in which the TPR forms the ordinate and FPR = 1–specificity is shown on the abscissa. The total area under this curve (AUC) is often taken as a measure of discriminating power of a procedure. Each pattern then forms a pair of values (FPR, TPR), with thousands of patterns furnishing a nonparametric or empirical ROC curve (Nahm, 2022). We generally build classes of FPR values and compute the average TPR in each class. This was implemented in a Pascal script.

**FIGURE 2:**
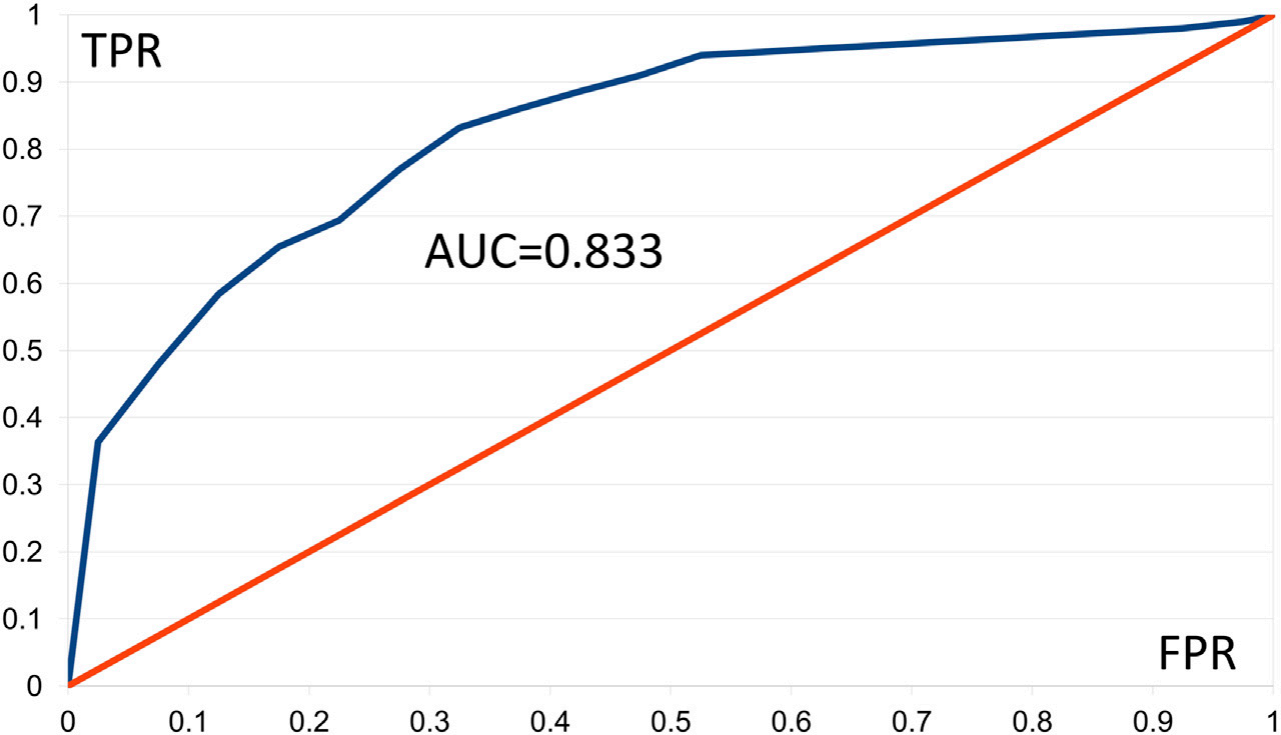
Empirical ROC curve resulting from the *Gpairs* analysis of the AMD dataset, based on the Fisher F1 test, the 100 best patterns, and 20 classes of FPR values. FPR = false positive rate, TPR = true positive rate, AUD = area under the curve.

We constructed ROC curves for the AMD data applying Fisher tests F1 and F2, using 20 classes of FPR values and 100 of the best patterns. The F1 and F2 tests furnished respective AUC values of 0.833 and 0.817. Figure 2 shows the ROC curve based on the F1 test, in which presence of a pattern predicts “control.” Values of TPR ≥ 0.945 were linearly interpolated as no corresponding FPR values occurred. Results with AUC ≥ 0.8 are generally considered good (Nahm, 2022). These results support our comment in Section 3.1.2 that AMD shows aspects of a digenic trait.

## 5 Discussion

For larger datasets involving, for example, 1 million variants, the total number of genotype pairs to be tested is on the order of 10^12^. Depending on the number of individuals, such calculations can take considerable time. As a remedy, we propose that the variants be pruned to a much smaller set of independent variants. This may be achieved, for example, with the *plink* option, --indep 50 5 2, where the parameters are as given in the *plink* manual (https://zzz.bwh.harvard.edu/plink/dist/plink-doc-1.07.pdf). Applied to the PD dataset, the original number of 379,485 variants was whittled down to 106,056 (28% of 379,485) independent variants. Another, larger dataset (not discussed here) of 891,689 variants furnished 26% independent variants. Thus, only 0.26^2^ = 6.8% of the original number of variant pairs need to be evaluated. Nonetheless, it will be time-consuming to apply our approach to very large datasets, although progress in computer technology tends to be fast.

Our prototype of two algorithms for exploring all pairs of variants and genotypes is already rather efficient on desktop computers with multiple CPUs. Refinements of the Pascal code may increase efficiency further. Two aspects of this project will be addressed in the near future, statistical significance and different phenotypes. Permutation analysis would be helpful for estimating *p*-values properly corrected for multiple testing, but *n* permutations will essentially require an *n*-fold increase in execution times. Also, we plan to expand case-control phenotypes to quantitative observations including covariates. One possibility to consider will be a logistic regression model that includes presence and absence of patterns as covariates.

An important aspect of our methods is that missing genotypes are handled correctly, which is an issue rarely discussed in pattern analysis. Also, it is noteworthy that in each dataset tested, we found many genotype pairs more significant (on the scale of Fisher *p*-values) than the best genotype pair extracted from analyzing variant pairs.

Our use of empirical ROC curves represents a simple introduction into this decision aspect of genotype patterns. Various extensions are conceivable and will be pursued at a later date.

## Data Availability

The original contributions presented in the study are included in the article/Supplementary Material, further inquiries can be directed to the corresponding author.
